# GeneNoteBook, a collaborative notebook for comparative genomics

**DOI:** 10.1093/bioinformatics/btz491

**Published:** 2019-06-14

**Authors:** Rens Holmer, Robin van Velzen, Rene Geurts, Ton Bisseling, Dick de Ridder, Sandra Smit

**Affiliations:** 1 Laboratory of Molecular Biology, 6708PB Wageningen, The Netherlands; 2 Bioinformatics Group, Wageningen University, 6708PB Wageningen, The Netherlands

## Abstract

**Summary:**

Analysis and comparison of genomic and transcriptomic datasets have become standard procedures in biological research. However, for non-model organisms no efficient tools exist to visually work with multiple genomes and their metadata, and to annotate such data in a collaborative way. Here we present GeneNoteBook: a web based collaborative notebook for comparative genomics. GeneNoteBook allows experimental and computational researchers to query, browse, visualize and curate bioinformatic analysis results for multiple genomes. GeneNoteBook is particularly suitable for the analysis of non-model organisms, as it allows for comparing newly sequenced genomes to those of model organisms.

**Availability and implementation:**

GeneNoteBook is implemented as a node.js web application and depends on MongoDB and NCBI BLAST. Source code is available at https://github.com/genenotebook/genenotebook. Additionally, GeneNoteBook can be installed through Bioconda and as a Docker image. Full installation instructions and online documentation are available at https://genenotebook.github.io.

**Supplementary information:**

Supplementary data are available at *Bioinformatics* online.

## 1 Introduction

Browsing, querying and comparing large genomic and transcriptomic datasets are indispensable aspects of genomic research. In recent years the decrease in cost of sequencing DNA or RNA has unlocked the possibility to generate eukaryotic genome assemblies with limited effort. As a result, genome analysis has become a routine exercise for research groups working on non-model organisms. Annotated genome sequences with metadata are used to identify candidate genes that can be targeted in wet lab experiments. As an example, integrating information on ortholog groups, protein domains and gene expression levels can provide valuable information on a gene’s hypothetical function. For such integration, it is crucial to be able to browse, query and compare genomic data, and curate automated predictions. This should ideally be a collaborative effort between experimental and computational researchers, and should be an efficient process that requires minimal configuration.

Currently, no efficient tool exists to quickly query, browse and visualize genomic data. Whereas genome browsers such as JBrowse (Buels *et al.*, 2016; Skinner *et al.*, 2009) provide powerful visualizations, they are limited to positional queries and visualizations. As an extension to JBrowse, Apollo allows for the curation of gene structure models (Lee *et al.*, 2013). However, both JBrowse and Apollo are limited to single genomes. Additionally, genome browsers are not very suitable for the integration of various data types, such as gene expression levels and ortholog groups. Data warehouse systems, such as InterMine (Smith *et al.*, 2012), provide more powerful query options but are relatively difficult to configure and generally do not come with data visualization options. Previously, data warehouse systems like InterMine and genome browsers like JBrowse have been combined into custom one-off data portals for model organisms, such as Araport for *Arabidopsis thaliana* (Krishnakumar *et al.*, 2015), the Legume Information System for legumes (Gonzales *et al.*, 2005) or Wormbase for *Caenorhabditis elegans* and related nematodes (Stein *et al.*, 2001). However, setting up a custom data portal for each new genome is inefficient and time consuming. Additionally, it is currently not possible to collaboratively curate genomic metadata, for instance, by adding curator notes to genes.

To enable quick and intuitive browsing and querying of genomic data for newly sequenced organisms we have developed GeneNoteBook: a collaborative web-based notebook for comparative genomics. Our application is designed for comparative analysis of genomic data and collaborative annotation of predicted genes with expert knowledge, by integrating genome annotations, gene expression data and gene evolutionary relationships.

## 2 Features

GeneNoteBook provides users with two views on their genomic data: a spreadsheet-like gene table with customizable fields and queries to browse and visualize information for multiple genes from multiple genomes, and a gene page with all available information for any particular gene.

**Fig. 1.**
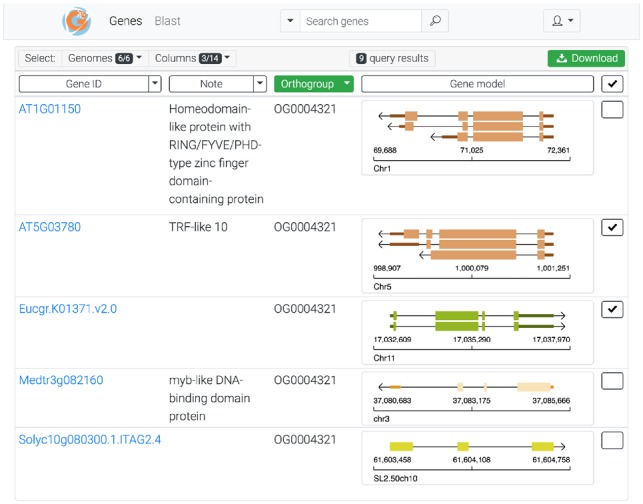
GeneNoteBook gene table view. This view shows genes in ortholog group OG0004321. Several genes have notes that indicate these genes are transcription factors. UTR regions vary, but most gene models have similar exon structures. Three genes have been selected for downloading

The gene table is designed such that users can intuitively query genes and visualize attributes of interest for query results. For example, users can quickly inspect the gene models of genes in an ortholog group (Fig. 1) and for the same genes inspect predicted protein domains or expression levels (Supplementary Fig. S1).

The gene page includes general information like chromosomal location, DNA sequence strand and any additional attributes like gene names or Gene Ontology terms (Supplementary Fig. S2). Through the user interface existing attributes can be curated, and new attributes can be added. This allows research groups to add names and update notes for their genes of interest in a collaborative fashion. Data provenance is provided by a version history that keeps track of all manual modifications, allowing them to be reversed if needed. In addition, the gene page offers visualizations of gene structure models, protein domains, ortholog group phylogenetic trees and gene expression levels.

A BLAST (Altschul *et al.*, 1990) service is implemented in GeneNoteBook to allow sequence-based searches against available genome annotations. BLAST results are linked to the gene table, which automatically allows querying and downloading data linked to the BLAST hits. Long running processes such as BLAST jobs are executed through a job queue, which automatically throttles the number of simultaneous jobs, keeps track of job progress and allows users to monitor jobs and remove or rerun them when necessary.

To allow for responsive browsing and querying of large genomic datasets, GeneNoteBook is implemented as a node.js webapp that dynamically renders HTML pages with SVG visualizations. All data are stored in the document-oriented NoSQL database MongoDB, with schemas tuned for efficient gene-centered queries. Examples of the visualization options, use cases and additional implementation details can be found in the Supplementary Data.

## 3 Conclusion

GeneNoteBook is specifically geared toward comparative genomics, since it is designed to store multiple genomes. We have successfully used GeneNoteBook for the comparison of several plants from the genera *Parasponia* and *Trema* to study the evolution of rhizobium symbiosis (van Velzen *et al.*, 2018). To demonstrate the potential of GeneNoteBook, a public instance hosting various plant genomes is available through the online documentation. These projects have demonstrated that GeneNoteBook is useful for both experimental biologists and bioinformatics researchers. This integrative approach facilitates studies of newly sequenced organisms compared with related organisms or well-annotated model organisms. Whereas our examples include plants, GeneNoteBook permits genomic data from any organism, even over large evolutionary distances. Additionally, GeneNoteBook offers several options for a smooth installation and configuration, such as a Bioconda (Grüning *et al.*, 2018) distribution and a Docker image. As such, GeneNoteBook has the potential to be used in a wide range of genome projects.

## Supplementary Material

btz491_Supplementary_Data

## References

[btz491-B1] Altschul S.F. et al (1990) Basic local alignment search tool. J. Mol. Biol., 215, 403–410. 2231712 10.1016/S0022-2836(05)80360-2

[btz491-B2] Buels R. et al (2016) JBrowse: a dynamic web platform for genome visualization and analysis. Genome Biol., 17, 66. 27072794 10.1186/s13059-016-0924-1 PMC4830012

[btz491-B3] Gonzales M.D. et al (2005) The Legume Information System (LIS): an integrated information resource for comparative legume biology. Nucleic Acids Res., 33, D660–D665. 15608283 10.1093/nar/gki128 PMC540082

[btz491-B4] Grüning B. et al (2018) Bioconda: sustainable and comprehensive software distribution for the life sciences. Nat. Methods, 15, 475–476. 29967506 10.1038/s41592-018-0046-7 PMC11070151

[btz491-B5] Krishnakumar V. et al (2015) Araport: the arabidopsis information portal. Nucleic Acids Res., 43, D1003–D1009. 25414324 10.1093/nar/gku1200 PMC4383980

[btz491-B6] Lee E. et al (2013) Web Apollo: a web-based genomic annotation editing platform. Genome Biol., 14, R93. 24000942 10.1186/gb-2013-14-8-r93 PMC4053811

[btz491-B7] Skinner M.E. et al (2009) JBrowse: a next-generation genome browser. Genome Res., 19, 1630–1638. 19570905 10.1101/gr.094607.109 PMC2752129

[btz491-B8] Smith R.N. et al (2012) InterMine: a flexible data warehouse system for the integration and analysis of heterogeneous biological data. Bioinformatics, 28, 3163–3165. 23023984 10.1093/bioinformatics/bts577 PMC3516146

[btz491-B9] Stein L. et al (2001) WormBase: network access to the genome and biology of *Caenorhabditis elegans*. Nucleic Acids Res., 29, 82–86. 11125056 10.1093/nar/29.1.82 PMC29781

[btz491-B10] van Velzen R. et al (2018) Comparative genomics of the nonlegume *Parasponia* reveals insights into evolution of nitrogen-fixing rhizobium symbioses. Proc. Natl. Acad. Sci. USA, 115, E4700–E4709. 29717040 10.1073/pnas.1721395115 PMC5960304

