# Supplementary material for: GeneNoteBook, a collaborative notebook for comparative genomics
Source: Bioinformatics. 2019 Jun 14;35(22):4779–81. doi: 10.1093/bioinformatics/btz491 (PMC6853645; doi:10.1093/bioinformatics/btz491)
Supplement: btz491_Supplementary_Data [file btz491_supplementary_data.zip › btz491-suppl-data/supplementary_data.pdf]

GeneNoteBook Supplementary Information

Table of content

1. Introduction
2. Features
   2.1. Searchable gene table with visualizations
   2.2. Single gene page with version history
   2.3. Visualizations
   2.4. Sequence search using BLAST
3. Use cases
   3.1. Visualizing gene expression of BLAST results
   3.2. Downloading protein sequences of all genes in an ortholog group
   3.3. Annotating orthologs of known genes
   3.4. Browsing differentially expressed genes
4. Implementation details
   4.1. Data types and file formats
   4.2. User accounts
   4.3. Administrator section
   4.4. Job queue
5. Installation options
6. Public instance

1. Introduction

GeneNoteBook is a web-based collaborative notebook for annotated genomes that facilitates intuitive browsing and querying of multiple eukaryotic genomes and their meta data, as described in the main text. This document provides more detailed descriptions and examples of GeneNoteBook's functionality than can be provided in the main text. For demonstration purposes, we use publicly available genomic and transcriptomic data of the model plant species *Medicago truncatula* (Young *et al.*, 2011) and *Arabidopsis thaliana* (Cheng *et al.*, 2017). The use cases are based on workflows from our previous work on *Parasponia andersonii*, a new model for studying the symbiotic relationship between rhizobium bacteria and plants (Van Velzen *et al.*, 2018).

2. Features

2.1 Searchable gene table with visualizations

Genome annotations loaded into a GeneNoteBook instance can be browsed through a dynamically rendered HTML table that combines queries with SVG data visualizations. Users can select genome annotation tracks for one or more genomes, and select gene attributes to be displayed as table columns. The table can be sorted and filtered on the selected attribute columns. Additionally, one of the following three data visualizations can be selected to be included in the table: **1.** a graphical representation of the gene model (As seen in Fig. 1 of the main text), **2.** protein domains as predicted by InterProScan (Jones *et al.*, 2014) (Suppl. Fig. 1a), or **3.** gene expression levels (Suppl. Fig. 1b). To facilitate intuitive

browsing and discovery of genes of interest, the gene table always starts with a view of all available data that can subsequently be narrowed down by constructing a query. This ensures that users need only limited prior knowledge to start browsing.

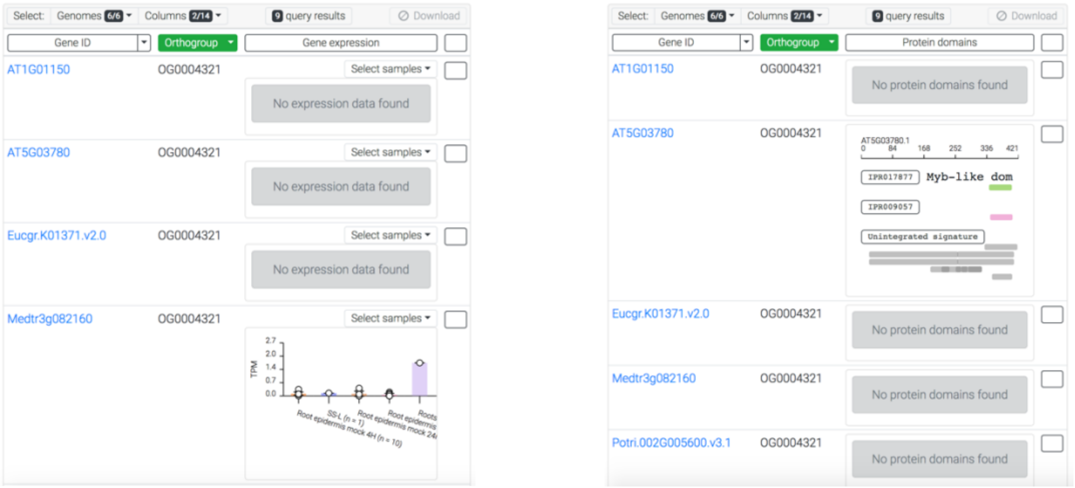

Figure 1. GeneNoteBook's gene table with SVG visualizations. Gene tables visualizing expression levels (left panel) and InterProScan predicted protein domains (right panel) for the same ortholog group (OG0004321) as in fig. 1 of the main text. By using the gene table, it is trivial to see what types of information are available for all hits that result from a query for a variety of organisms.

2.2 Single gene page with version history

For every gene in a GeneNoteBook instance, a comprehensive information page is rendered. The single gene page starts with a list of all gene attributes in the form of key-value pairs. Users with curator or administrator access (see Section 3.2 for user account types) can modify existing attributes, or add new attributes. This allows for the curation of automatically assigned protein product names, or addition of trivial names or notes to genes of interest. To prevent simultaneous conflicting edits to the same gene, once a user starts editing the attributes of a gene, the ability to edit the gene is locked for all other users. Once the edited fields are saved, the gene is unlocked and all changes are saved to a version history. To make sure a user cannot lock a gene forever the gene is automatically unlocked after 10 minutes of inactivity. A version history that is maintained in the database assures that all manual curations and additions can be tracked and reverted if needed.

In addition to the listed attributes, the single gene page contains a panel with the coding sequence of all transcripts of the gene and visualizations for the gene model, predicted protein domains, gene expression levels and a phylogenetic tree for the corresponding ortholog group.

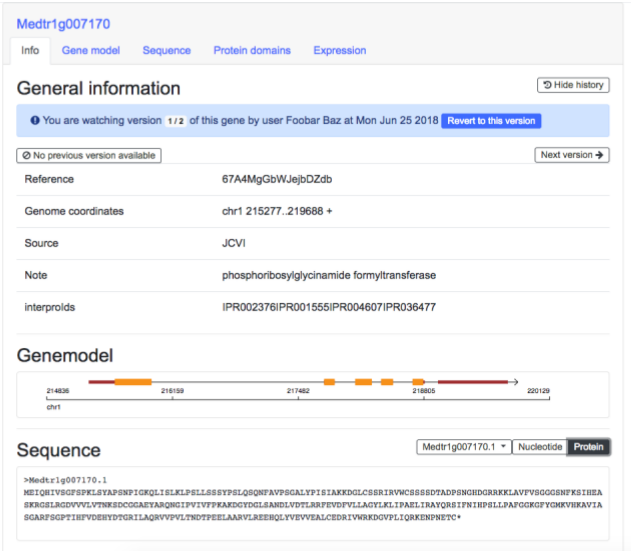

Figure 2: Top part of the gene page highlighting the *Medicago truncatula* gene Medtr1g007170 with version history. The blue bar at the top highlights that this gene has been edited in the past and currently has two versions. Curator and administrator users can decide to revert the changes by clicking the 'Revert to this version' button.

2.3 Visualizations

GeneNoteBook dynamically renders SVG visualizations for various data types and analysis results. Since GeneNoteBook's minimally required input data is an annotated genome sequence, the gene structure model visualization is always available. Visualizations for predicted protein domains, ortholog group phylogenetic trees and gene expression levels become available once the appropriate data (see Supplementary Table 1) are loaded into GeneNoteBook.

The gene model visualization provides a graphical representation of the transcripts and exons of a gene. Coding sequence exons are rendered larger than UTR exons to accentuate the difference. Exons or mRNAs can be clicked to display exon- or mRNA-specific information, such as coordinates and IDs, in a pop up window.

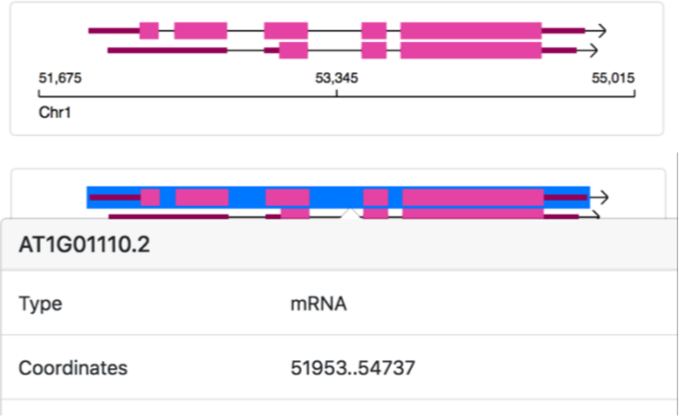

Figure 3: Gene model visualization of the Arabidopsis thaliana gene AT1G01110 with two alternative splice forms. The general genomic location of this gene is on chr1, starting at nucleotide 51,675 and ending at nucleotide 55,015 (top panel). The exact coordinates of each exon or mRNA can be accessed through a popup window that opens when an exon or intron is clicked or hovered. In this case the full mRNA interval has been clicked, showing that it ranges from 51,953 to 54,737 (bottom panel). Black lines indicate introns. Narrow purple bars indicate UTR regions. Wider pink bars indicate coding sequence exons.

Protein domains as predicted by InterProScan (Jones *et al*., 2014) are displayed sorted by Interpro domain type, with their Interpro ID and short description. Protein domains can be clicked to display additional information in a pop up window.

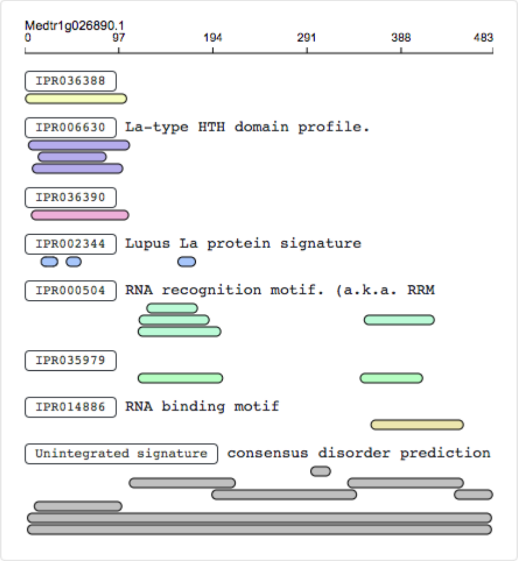

Figure 4: InterProScan protein domain prediction visualization for the first splice form of the *Medicago truncatula* gene Medtr1g026890. The axis at the top of the visualization represents the length of the protein in amino acids. Protein domains are sorted and colored by their Interpro annotation. More information on the source of the prediction and other information provided by InterProScan is available in a popup window that can be accessed by hovering or clicking a domain.

Ortholog group membership is visualized as a phylogenetic tree. Other genes in the phylogenetic tree are automatically turned into hyperlinks if they are included in the GeneNoteBook instance, which greatly simplifies navigating between various genes within an ortholog group. By default the genes in the tree are labelled with their gene ID and, if available, their name. Genes are colored based on the genome in which they are found.

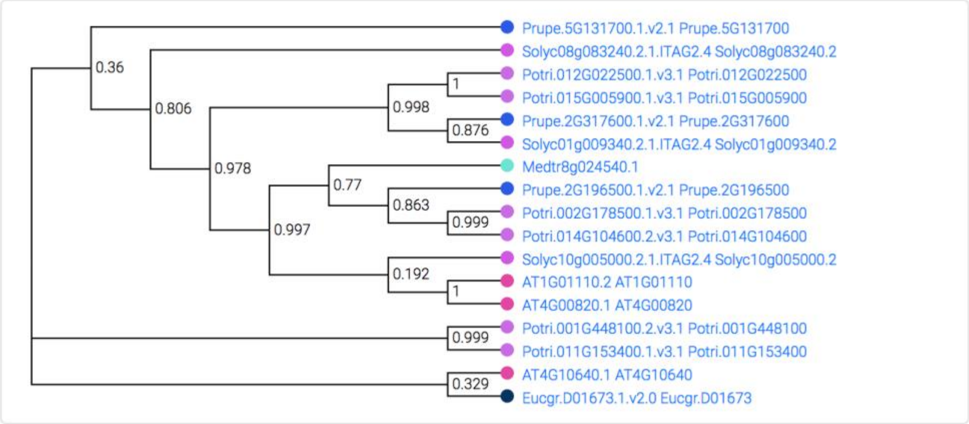

Figure 5: Phylogenetic tree visualization for an ortholog group that contains genes from several plant species. GeneNoteBook presents genes that can be linked by their gene ID as hyperlinks to the single gene page of that gene. Additionally, internal nodes display confidence values that are parsed from the Newick formatted tree file used as input. Tip nodes are colored according to the corresponding organism: *Prunus persica* (blue), *Solanum lycopersicum* (ligh purple), *Populus trichocarpa* (dark purple), *Medicago truncatula* (teal), *Arabidopsis thaliana* (pink), *Eucalyptus grandis* (black). This ortholog group was identified by OrthoFinder (Emms and Kelly, 2015), the phylogenetic tree was constructed using the neighbor joining algorithm (Saitou and Nei, 1987) on a MAFFT (Katoh and Standley, 2013) multiple sequence alignment.

GeneNoteBook can visualize gene expression quantified from RNA sequencing experiments as bar plots with error-bars representing the standard error. Additionally, individual data points are displayed as open circles. Transcriptome samples can be grouped in replica groups either upon loading the data, or in the Admin panel. Y-axis limits are automatically calculated from the data and a drop down menu allows users to select which experiments they want displayed.

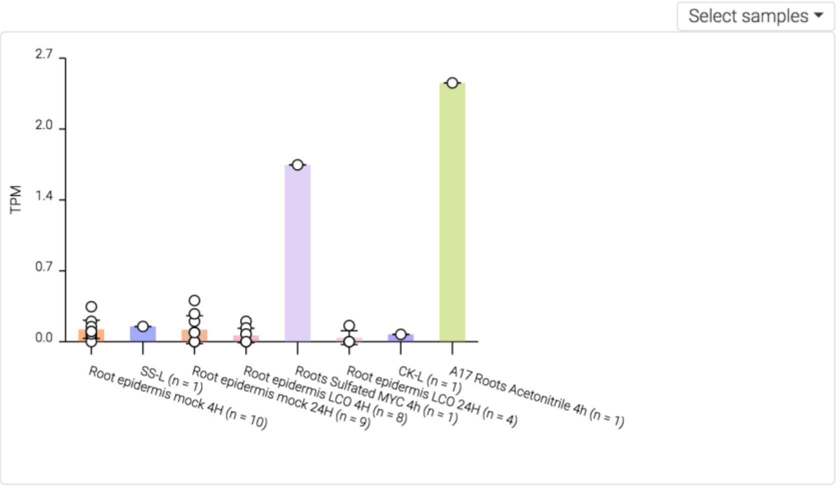

Figure 6: GeneNoteBook SVG barplot of expression levels. Quantified gene expression is shown for the *Medicago truncatula* gene Medtr3g082160 in several publicly available transcriptome datasets (BioProjects PRJNA283476 and PRJNA255840). Experiments with multiple replicates are grouped together into replica groups, bars are automatically colored according to replica group. Labels per bar indicate the name of the replica group and automatically add the number of data points. Individual data points are plotted over a bar that represents the mean expression level, with error bars indicating the standard error. Gene expression was quantified using kallisto (Bray *et al.*, 2016). TPM = transcripts per million.

2.4 Sequence search using BLAST

To allow sequence-based searching of gene models, GeneNoteBook implements a wrapper around BLAST (Altschul *et al*., 1990). Users can submit fasta formatted DNA or protein sequences to BLAST against GeneNoteBook's genome annotations. BLAST jobs are handled by GeneNoteBook's job queue such that the server will not be flooded with blast jobs and results can be stored. Results are presented as an SVG visualization visualization showing the blast hits relative to the query sequence and a list with hits sorted by E-value.

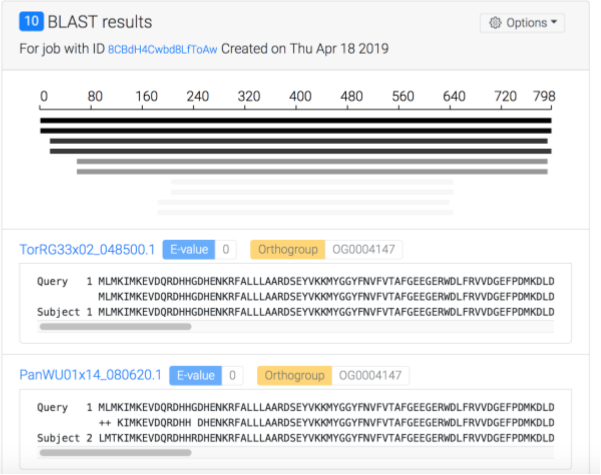

Figure 7: BLAST result visualization. Every BLAST job has a unique ID with a corresponding URL so that BLAST jobs can be retrieved at a later moment. The figure depicts the location of the BLAST hits relative to the query, with a darker color indicating a higher bit-score. Below the visualization all hits are listed with their corresponding sequence alignments and a hyperlink to the corresponding gene. The options menu in the top right corner allows users to select various visualizations for the BLAST hits, including gene expression levels and protein domain predictions. Additionally, users can send the results to the gene table interface to make additional queries on the BLAST hits, or to download the corresponding data.

3. Use cases

These use cases are based on workflows from our previous work on *Parasponia andersonii*, a tropical tree for which we newly sequenced and annotated its genome (Van Velzen *et al*., 2018). We have populated a GeneNoteBook server with the genomes and protein-coding gene annotations of *Medicago truncatula*, *Arabidopsis thaliana* and *Parasponia andersonii*. Additionally, we have added ortholog group information that includes these species, as well as protein domain predictions quantified gene expression levels for *P. andersonii*.

3.1 Visualizing gene expression of BLAST results

Whenever a new gene is discovered to be involved in rhizobium symbiosis in a species that we have not included in our ortholog groups (i.e. *Lotus japonicus*), we use the GeneNoteBook BLAST functionality to quickly determine the *P. andersonii* homologs and their expression levels in various tissues.

Step 1. Obtain the original nucleotide/protein sequence for the gene of interest

Step 2. BLAST the gene sequence against the *P. andersonii* genome annotation

Step 3. Select the Gene Expression visualization from the options menu in the BLAST results section

Step 4. Optionally, send the BLAST results to the Gene Table section for additional queries

3.2 Downloading protein sequences of all genes in an ortholog group

We predominantly use ortholog group phylogenetic trees constructed with the neighbor joining algorithm in our GeneNoteBook. Whenever we doubt the accuracy of a phylogenetic tree, we download the protein sequences of all genes in the phylogenetic tree and run more sophisticated tree reconstruction methods based on maximum likelihood or Bayesian inference.

Step 1. For a gene of interest, collect the orthology group ID

Step 2. Select all genes that belong to the ortholog group in the Gene Table by enabling the ortholog group column from the 'Select Column' menu and pasting the ortholog ID in the 'Filter → Equals' box

Step 3. Check the 'Select All' checkbox in the top right of the Gene Table

Step 4. Click 'Download'

Step 5. Select 'Sequences' in the download menu

Step 6. Download the protein sequences of these genes

3.3 Annotating orthologs of known genes

We started our project on the newly sequenced and annotated genome of *P. andersonii* by taking genes of *M. trucatula* that are known to be involved in rhizobium symbiosis, and finding their *P. andersonii* orthologs. We subsequently named the *P. andersonii* genes through the GeneNoteBook editing functionality. Naming a gene is a quick way of ensuring easy retrieval of the gene at later stages. In this fashion, we named ~2000 genes with a group of six people over a period of a few weeks.

Step 1. Search for an *M. truncatula* gene that is involved in symbiosis

Step 2. Interpret the ortholog group phylogenetic tree to identify the *P. andersonii* ortholog

Step 3. Browse to the *P. andersonii* ortholog and add a name attribute

3.4 Browsing differentially expressed genes

In a subsequent experiment, we determined gene expression levels in uninoculated roots and in root nodules that contain rhizobium bacteria. We determined significant differential expression using external tools, and used GeneNoteBook to identify what these differentially expressed genes are. Specifically, we looked for known symbiosis genes by querying on the names from example 3.1.

Step 1. Determine differential expression, and obtain a list of gene IDs for genes that are differentially expressed between uninoculated roots and root nodules

Step 2. Add a GeneNoteBook query for these gene IDs by pasting a list of new-line separated gene IDs in the query box

Step 3. Add a GeneNoteBook query for genes that have the Name attribute by first enabling the Name attribute in the 'Select Columns' menu and subsequently selecting 'Filter → Present' in the Name attribute column header.

4. Implementation details

GeneNoteBook is a meteor.js web app consisting of a node.js server and a JavaScript browser client. Meteor.js was chosen for three main reasons. First, meteor.js apps are designed to work with the document-oriented NoSQL database MongoDB. The NoSQL document model of MongoDB provides a flexible data model. This is convenient, since the types of data used in GeneNoteBook are often not easily represented in tabular form (i.e. a gene model is typically represented as a directed acyclic graph of exons). Second, meteor.js comes with several default functionalities such as a user account system and a job-queue system. Third, since one of the key features of GeneNoteBook is the ability to edit gene attributes, GeneNoteBook uses the meteor.js publication-subscription system over WebSocket to be

able to actively push changed data to connected clients. This ensures that users always receive up-to-date data and allows GeneNoteBook to function as a real-time dynamic web application. For dynamic rendering, both for the HTML user interface and the SVG visualizations, GeneNoteBook uses React.js.

4.1 Data types and file formats

GeneNoteBook integrates queries and visualizations for various data types (Supplementary Table 1). The minimal amount of required data needed to run GeneNoteBook is a reference genome sequence in FASTA format, and a protein coding gene annotation in GFF3 format. Since GeneNoteBook uses the GFF3 ID attribute to uniquely identify genes, *every gene interval in the GFF3 file must have a unique ID.* Commonly used gene prediction algorithms generally fulfill this requirement, and GeneNoteBook is designed to work on result files from Maker (Campbell *et al.*, 2014), EvidenceModeler (Haas *et al.*, 2008) and BRAKER (Hoff *et al.*, 2016).

Table 1: GeneNoteBook data types and file formats

**Data type**	**File format**	**Required?**
Reference genome sequence	.fasta	yes
Protein coding gene annotation	.gff3	yes
InterProScan protein domain prediction	.gff3 (InterProScan)	no
Ortholog group phylogenetic tree	.newick[1] (OrthoFinder)	no
Transcriptome quantification	.tsv[2] (Kallisto)	no

4.2 User accounts

To handle data permissions and allow users to save BLAST results GeneNoteBook uses a minimalistic user account system. By default, four access levels are used to distinguish between users: 1) registered, 2) user, 3) curator and 4) administrator. If someone registers an account they become ‘registered’, but have no access to any data yet. Once an administrator changes the account to ‘user’, (s)he can start browsing data. Curators are allowed to make changes to gene attributes. Administrators have full access to all data, can assign permissions to other users and have access to the ‘Admin settings’ section of the GeneNoteBook site, where they can configure GeneNoteBook.

Administrator users get access to the Admin panel of GeneNoteBook, which can be used to monitor and configure user profiles, permissions, BLAST databases, gene attributes, transcriptome datasets and the job queue (Fig. 8).

[1] The Kallisto tab delimited file format has the following five fields: transcript ID, transcript length, effective transcript length, est counts, transcripts per million. The first line is a header and is skipped during parsing. Only the transcript ID, est counts, and transcripts per million fields are parsed.

[2] Every ortholog group phylogenetic tree should be in a separate file. The file name is treated as the orthogroup identifier.

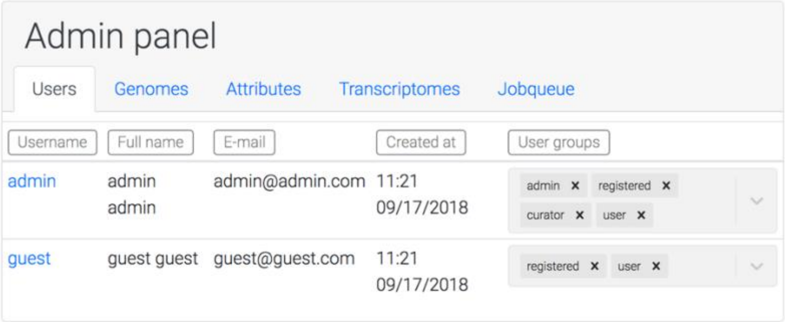

Figure 8: The administrator section of GeneNoteBook. This section gives an overview of registered accounts and user permissions. Additionally, the accessibility of loaded genomes, transcriptomes and annotation tracks can be configured, and current and old jobs in the job queue can be monitored and configured.

4.3 Job queue

To execute long-running processes like BLAST and preparing data for downloading, GeneNoteBook uses a job queue system. The job queue can be configured to process up to a specified maximum number of jobs in parallel. This ensures that the machine hosting GeneNoteBook will not be overloaded, but also allows upscaling for GeneNoteBook instances that are heavily used. The job queue is implemented in the database such that no external configuration is necessary. Additionally, by storing job status and results in the database, jobs can be tracked in real-time using GeneNoteBook's reactive publication/subscription system. To prevent old jobs from accumulating, by default a cleaning job runs every 20 minutes to remove jobs that are older than one month.

5. Installation options

GeneNoteBook depends on node.js, MongoDB and BLAST. To allow several varieties of dependency management and configuration, GeneNoteBook can be installed and run in three ways: 1) download a release tarball from github, 2) install through bioconda (Grüning *et al*., 2018) or 3) run as a Docker container. When downloading the release tarball from github, the dependencies must be installed manually and the MongoDB daemon must be started manually before GeneNoteBook can start. When using the bioconda distribution, all dependencies are installed by bioconda, but again the MongoDB daemon must be started manually. When using the Docker distribution, all dependencies are installed in the GeneNoteBook Docker container and a separate Docker container for the MongoDB daemon is started automatically. More information on how to configure a GeneNoteBook deployment can be found in the online documentation at http://genenotebook.github.io/.

6. Public instance

To display the full potential of GeneNoteBook, an example instance with several public datasets is available at http://bioinformatics.nl/genenotebook. Users can login to this public instance with username *guest* and password *guestguest*.

References

Altschul, S. F., *et al.* (1990). Basic local alignment search tool. *J. Mol. Biol.*, **215**(3), 403–410.

Bray, N. L., *et al.* (2016). Near-optimal probabilistic RNA-seq quantification. *Nat. Biotechnol.*, **34**(8), 888.

Campbell, M. S., *et al.* (2014). MAKER-P: a tool kit for the rapid creation, management, and quality control of plant genome annotations. *Plant Physiol.*, **164**(2), 513–524.

Cheng, C.-Y., *et al.* (2017). Araport11: a complete reannotation of the Arabidopsis thaliana reference genome. *Plant J.*, **89**(4), 789–804.

Emms, D. M. and Kelly, S. (2015). OrthoFinder: solving fundamental biases in whole genome comparisons dramatically improves orthogroup inference accuracy. *Genome Biol.*, **16**, 157.

Grüning, B., *et al.* (2018). Bioconda: sustainable and comprehensive software distribution for the life sciences. *Nat. Methods*, **15**(7), 475–476.

Haas, B. J., *et al.* (2008). Automated eukaryotic gene structure annotation using EVidenceModeler and the Program to Assemble Spliced Alignments. *Genome Biol.*, **9**(1), R7.

Hoff, K. J., *et al.* (2016). BRAKER1: Unsupervised RNA-Seq-Based genome annotation with GeneMark-ET and AUGUSTUS. *Bioinformatics*, **32**(5), 767–769.

Jones, P., *et al.* (2014). InterProScan 5: genome-scale protein function classification. *Bioinformatics*, **30**(9), 1236–1240.

Katoh, K. and Standley, D. M. (2013). MAFFT multiple sequence alignment software version 7: improvements in performance and usability. *Mol. Biol. Evol.*, **30**(4), 772–780.

Saitou, N. and Nei, M. (1987). The neighbor-joining method: a new method for reconstructing phylogenetic trees. *Mol. Biol. Evol.*, **4**(4), 406–425.

Van Velzen, R., *et al.* (2018). Comparative genomics of the nonlegume Parasponia reveals insights into evolution of nitrogen-fixing rhizobium symbioses. *Proc. Natl. Acad. Sci.*, **115**(20), E4700-E4709.

Young, N. D., *et al.* (2011). The Medicago genome provides insight into the evolution of rhizobial symbioses. *Nature*, **480**(7378), 520–524.
